# Phase IV, open-label, randomized study of low-dose recombinant human follicle-stimulating hormone protocols for ovulation induction

**DOI:** 10.1186/1477-7827-12-52

**Published:** 2014-06-18

**Authors:** Gamal I Serour, Mohamed Aboulghar, Awatef Al Bahar, Jean-Noel Hugues, Khaled Esmat

**Affiliations:** 1 Department of Obstetrics and Gynaecology and International Islamic Center for Population Studies and Research, Al Azhar University, Cairo, Egypt; 2 Egyptian IVF Center, Maadi, Cairo, Egypt and Department of Obstetrics and Gynecology, Cairo University, Cairo, Egypt; 3 Dubai Gynecology and Fertility Center, Dubai, UAE; 4 Department of Obstetrics and Gynaecology, Center for Reproductive Medicine, Jean Verdier Hospital, University Paris XIII, Paris, France; 5 Department of Medical Affairs, Merck Serono Middle East FZ – LLC, Dubai, UAE; 6 Current address: Department of Medical Affairs, Genzyme Intercontinental Region, Dubai, UAE

**Keywords:** Anovulatory infertility, Recombinant human follicle-stimulating hormone, Low-dose protocol, Ovulation induction

## Abstract

**Background:**

This Phase IV, open-label, multicentre, randomized study (MEnTOR) compared two low-dose recombinant human follicle-stimulating hormone (r-hFSH) protocols for ovulation induction.

**Methods:**

This study was conducted in six Middle Eastern countries between March 2009 and March 2011. Eligible women (18–37 years), with World Health Organization Group II anovulatory infertility, were randomized to receive r-hFSH (starting daily dose: 75 IU) as a chronic low-dose (CLD) (37.5 IU dose increase on Day 14) or low-dose (LD) (37.5 IU dose increase on Day 7) protocol if no follicles were ≥10 mm. The maximum r-hFSH daily dose permitted was 225 IU/day. The total length of ovarian stimulation could not exceed 35 days, unless ultrasound assessment suggested imminent follicular growth and maturation. Patients underwent only one treatment cycle. Primary endpoint: incidence of mono-follicular development. Secondary endpoints included: stimulation duration and rates of bi-follicular development; human chorionic gonadotrophin administration rate; clinical pregnancy; and cycle cancellation (owing to inadequate response). Adverse events (AEs) were recorded. The primary efficacy analysis was performed using data from all patients who received at least one dose of correct study medication, had at least one efficacy assessment, and no protocol violations at treatment start (CLD group, *n* = 122; LD group, *n* = 125).

**Results:**

Mono-follicular development rates (primary endpoint) were similar in both groups (CLD: 56.6% [69/122] versus LD: 55.2% [69/125], p = 0.93; primary efficacy analysis population). Similarly, there were no significant differences between groups in bi-follicular development, clinical pregnancy or cycle cancellation (inadequate response) rates. In patients who received human chorionic gonadotrophin injections, the mean duration of stimulation was 13.7 days in the CLD group and 12.9 days in the LD group. Clinical pregnancy rates for those patients who received an hCG injection were similar in both groups (CLD: 20.2% [19/94] versus LD: 19.8% [18/91], p = 0.94; primary efficacy analysis population). Most AEs were mild in severity. Only one case of ovarian hyperstimulation syndrome was reported (mild; CLD group).

**Conclusions:**

Efficacy and safety outcomes were similar for the two protocols.

**Trial registration:**

Clinicaltrials.gov NCT01081626.

## Background

Ovulatory disorders account for approximately 30% of all cases of infertility [1]. World Health Organization (WHO) Group II anovulatory infertility is the most common form of ovulatory dysfunction and is characterized by asynchronous gonadotrophin production with follicle-stimulating hormone (FSH) and oestradiol (E_2_) levels within the normal range. A large proportion of women with WHO Group II anovulatory infertility have polycystic ovary syndrome [2].

First-line therapy for WHO Group II anovulatory infertility is usually clomiphene citrate (CC) [3,4]. However, a substantial proportion (approximately 40%) of women with WHO Group II anovulatory infertility fail to conceive following CC therapy [5]. Such patients may benefit from gonadotrophin therapy to stimulate follicle development and induce ovulation [1,4,6-9]. Ovulation induction (OI), however, may be associated with the serious complications of ovarian hyperstimulation syndrome (OHSS) and multiple pregnancy [9-11].

The amount of exogenous FSH required to induce follicular development (the so-called FSH threshold) is highly variable among individuals [12-16]. This is particularly important for women with WHO Group II anovulatory infertility and polycystic ovarian morphology [5], as the ovaries are extremely sensitive to gonadotrophin stimulation [8].

Chronic low-dose (CLD) step-up FSH protocols have been developed so that the lowest effective dose of FSH can be used to achieve the objective of mono-follicular development [5,16,17]. The classic CLD regimen involves a low daily starting dose (usually 75 IU) for 14 days and, if necessary, the FSH dose is increased in small increments (37.5 IU), at intervals of no fewer than 7 days, until follicular development is initiated [8]. Combined data from 11 studies indicate that such CLD protocols result in a high mono-ovulation rate (69% of cycles) and low multiple pregnancy and OHSS rates (5.7% and 0.14% of cycles, respectively) [8].

A modified protocol has also been developed and utilized by some clinicians in an attempt to shorten treatment schedules and reduce costs. In this so-called low-dose (LD) protocol, the starting dose of FSH (75 IU) is maintained for only 7 days before small incremental dose increases are permitted [8]. However, evaluation of such LD protocols comprises only one small single-centre study (*n* = 50), which found that although the duration of FSH stimulation was shorter, the risk of multi-follicular development was greater than with CLD protocols [8].

Technological advances allow recombinant human (r-h) FSH (follitropin alfa; GONAL-f^®^; Merck Serono S.A.– Switzerland, a subsidiary of Merck KGaA, Darmstadt, Germany) to be filled by mass (FbM) rather than by conventional bioassay [18]. This results in high batch-to-batch consistency and allows precise dosing of r-hFSH (follitropin alfa), which is likely to be particularly beneficial when using OI protocols with small dose increments. Indeed, it is possible that the use of r-hFSH (follitropin alfa) FbM may reduce the individual variability in ovarian response [19]. In turn, this may result in measurable differences in outcomes between LD and CLD protocols.

This article reports the findings of the first randomized, multicentre study to compare the efficacy and safety of CLD versus LD protocols for OI using r-hFSH (follitropin alfa FbM). The primary objective of this post-marketing study was to investigate the optimization of r-hFSH treatment in patients with chronic anovulation.

## Methods

### Study design

This randomized, multicentre, open-label, Phase IV study (Middle East Trial for Ovulation induction Responders [MEnTOR]; NCT01081626) was conducted between March 2009 and March 2011 in six Middle Eastern countries (Egypt, Iran, Kuwait, Lebanon, Saudi Arabia and United Arab Emirates). Ten sites were originally involved, with three additional sites later added because of slow recruitment.

The study was performed in accordance with the Declaration of Helsinki, the International Conference on Harmonisation Harmonised Tripartite Guideline for Good Clinical Practice and all applicable regulatory requirements - see Additional file 1. Independent ethics committee approval was obtained at each study centre. Written informed consent was obtained from all patients prior to study start.

### Study population

Women aged 18–37 years with WHO Group II anovulatory infertility (defined as a menstrual cycle duration >35 days or regular cycles with luteal-phase progesterone [P4] levels <10 nmol/mL) and who wished to conceive were eligible for inclusion in the study. Other key inclusion criteria were: body mass index (BMI) >20 to ≤32 kg/m^2^; spontaneous or CC-induced menses, or a positive progestin-induced withdrawal bleed within the previous year; normal uterine cavity and ≥1 patent fallopian tube; early follicular-phase FSH and prolactin levels within the normal range; total antral follicle count ≥10 (follicles of ≥2 to <11 mm in diameter in both ovaries); and a male partner with sperm considered normal based on local standards.

Patients who had received CC or gonadotrophins within 1 month of screening were excluded from participation. Other key exclusion criteria were: ovarian enlargement or ovarian cyst (unrelated to polycystic ovary syndrome); uterine fibroids; gynaecological bleeding of unknown aetiology; history of ≥3 miscarriages, extra-uterine pregnancy or severe OHSS (classified according to Royal College of Obstetricians and Gynaecologists criteria) [20]; clinically relevant systemic condition (e.g. insulin-dependent diabetes); and medical conditions that in the investigator’s opinion could prevent an effective response to treatment (e.g. primary ovarian failure, malformations of the reproductive organs incompatible with pregnancy) or affect the absorption, distribution, metabolism or excretion of the study drug.

### Treatments and interventions

The study comprised a screening visit (between Days 7 and 28 of the cycle, before the patient was assigned to treatment) followed by pre-stimulation, stimulation and post-stimulation periods. Patients underwent only one treatment cycle.

FSH, E_2_, luteinizing hormone and prolactin levels and antral follicle count (determined by transvaginal ultrasound [TVUS]) were measured at the screening visit.

**Figure 1:**
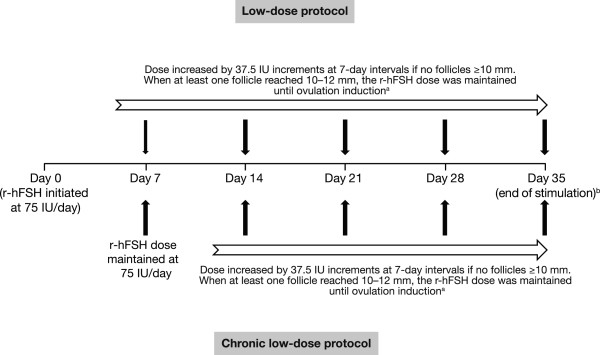
**Treatment protocols. **^a^When the leading follicle reached ≥17 mm and no more than two follicles had reached >14 mm, a single dose of hCG was injected to trigger ovulation. ^b^Duration of ovarian stimulation could not exceed 35 days, unless ultrasound assessment suggested imminent follicular growth and maturation. The maximum permitted daily dose of r-hFSH was 225 IU. Ovarian response was monitored by TVUS every 7 days until a follicle of ≥10 mm was observed, and then scheduled according to local practice. hCG = human chorionic gonadotrophin; IU, international units; r-hFSH = recombinant human follicle-stimulating hormone; TVUS, trans-vaginal ultrasound.

Following a negative pregnancy test, patients were randomized (1:1) to receive daily r-hFSH (follitropin alfa FbM) administered subcutaneously according to either a CLD or an LD protocol. Treatment allocation was determined by a computer-generated randomization list and balanced in a 1:1 ratio within each site. Investigators received randomization codes in individual sealed and numbered envelopes. At randomization, the investigator opened the envelope with the next available number to reveal the allocated treatment protocol, which was disclosed to both patients and investigators. Following randomization, study visits occurred weekly up to Day 35 (end of stimulation).

r-hFSH (follitropin alfa) was supplied in a pre-filled pen for injection (the GONAL-f^®^ Revised Formulation Female pen; EMD Serono, Inc., Rockland, MA, USA [a subsidiary of Merck KGaA, Darmstadt, Germany]) in three dose presentations (300, 450 or 900 IU). Administration of r-hFSH was initiated by physicians but (depending on the study centre) subsequent doses could be self-injected by patients; details of self-administered injections were recorded by the patient on a diary card.In the CLD protocol, r-hFSH was initiated at a daily dose of 75 IU with an increase of 37.5 IU on Day 14 if no follicles ≥10 mm were observed. In the LD protocol, r-hFSH was initiated at a daily dose of 75 IU with an increase of 37.5 IU on Day 7 if no follicles ≥10 mm were observed (Figure 1). In both protocols, subsequent dose increases were possible using 37.5 IU increments at 7-day intervals depending on ovarian response. The maximum permitted daily dose of r-hFSH was 225 IU. The total duration of ovarian stimulation could not exceed 35 days, unless ultrasound assessment suggested imminent follicular growth and maturation.

The ovarian response was monitored closely by TVUS every 7 days until a follicle of at least 10 mm in diameter was observed, and thereafter according to local practice. When at least one follicle reached 10–12 mm in diameter, the r-hFSH dose was maintained until the leading follicle reached ≥17 mm and no more than two follicles had reached >14 mm. A single dose of human chorionic gonadotrophin (hCG) was then injected to trigger ovulation. Either r-hCG 250 μg (Ovidrel^®^; EMD Serono, Inc.) administered subcutaneously or urine-derived hCG 5000 IU administered intramuscularly could be used. Intercourse was recommended on the day of, and the day after, hCG administration; intrauterine insemination could be performed using cryopreserved semen if the partner was unavailable. Patients considered to have an under-response (failure of at least one follicle to reach 10–12 mm in diameter) or an over-response (more than three follicles of >14 mm in diameter and/or E_2_ levels >900 pg/mL) after 35 days of r-hFSH stimulation were withdrawn from the study.

Serum P4 levels were measured 5–7 days and/or 8–10 days after hCG administration. Ovulation was assumed if mid-luteal P4 levels were ≥9.4 ng/mL (30 nmol/L) and/or pregnancy was achieved. A serum beta hCG pregnancy test was performed 15–20 days after hCG administration (if menstruation did not occur). Clinical pregnancy was confirmed by the presence of at least one foetal sac on TVUS 35–42 days after hCG administration.

### Outcomes

#### Efficacy outcomes

The primary efficacy endpoint was the incidence of mono-follicular development, defined as the development of only one follicle ≥17 mm in diameter (and ≤2 other intermediate [>14 mm] follicles) on or before Day 35 of stimulation.

Secondary endpoints were the incidence of bi-follicular development (defined as the proportion of patients developing two follicles ≥17 mm in diameter and ≤1 other intermediate [>14 mm] follicle); incidence of multi-follicular development (more than three follicles >14 mm in diameter); hCG administration rate; clinical pregnancy rate; rate of cycle cancellation (defined as the proportion of patients with cycle cancellation owing to an inadequate response to stimulation [i.e. failure of at least one follicle to reach ≥17 mm in diameter]); duration of r-hFSH stimulation before hCG administration; and total r-hFSH dose required.

The incidence of bi-follicular development was added after the protocol was finalized to better evaluate the two treatment protocols.

#### Safety outcomes

Safety endpoints included the incidence and severity of adverse events (AEs; including OHSS) and local tolerability of r-hFSH administration. AEs were recorded until at least 15–20 days after hCG administration (or 20–30 days after the last dose of r-hFSH if the patient was withdrawn prior to hCG administration). AEs that occurred >30 days after the last dose of r-hFSH were not recorded. The severity of AEs and their relationship to the study drug was determined by the investigator. Serious AEs and medically relevant ongoing/unknown-outcome AEs were followed-up until resolution or stabilization.

Multiple pregancy (confirmed by the presence of more than one foetal sac on TVUS 35–42 days after hCG administration) was also recorded as a safety endpoint. Rates of multiple pregancy and miscarriage are only presented for patients who received hCG injections.

### Statistical methods

The null hypothesis was that there was no difference in the proportion of patients achieving mono-follicular growth in each treatment group.

Determination of the target sample size was based on a single analysis of the primary endpoint using the Chi-square test and applying a two-sided 5% alpha level of significance. A mono-follicular development rate of 69% was reported in a previous study using a step-up urine-derived hFSH protocol with a starting dose of 75 IU [8]. A sample size of 134 evaluable patients per treatment group would be required to achieve 80% power to detect a 15% difference in the rate of mono-follicular development between groups, assuming a true rate of 70% in the LD protocol group. Enrolment of 150 women per treatment group was planned to allow for withdrawals and non-evaluable data.

The primary efficacy analysis was performed using data from all patients who received at least one dose of the correct study medication, had at least one efficacy assessment, and no protocol violations at the start of treatment. Secondary supportive analyses were performed using the per-protocol (PP) population (patients from the primary efficacy analysis population who received all doses of study medication, excluding patients with protocol deviations during the course of treatment). The safety population included all patients who received at least one dose of study medication and had one follow-up visit.

The primary and secondary efficacy endpoints were analysed using the Chi-square test, with the difference between treatment groups analysed using a 95% confidence interval, and a p-value was provided. Analysis of variance was used to evaluate the duration of stimulation and the dose of r-hFSH required.

## Results

### Patient population

**Figure 2:**
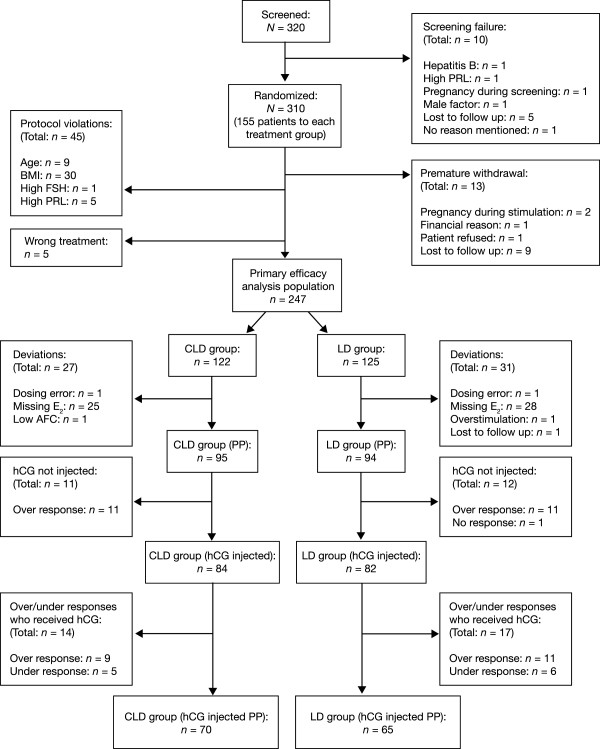
**Patient disposition.** Patients were considered to have had an under-response if there was failure to develop at least one follicle of 10–12 mm in diameter after 35 days of r-hFSH stimulation. Patients were considered to have had an over-response if they developed more than three follicles of >14 mm in diameter and/or their E_2_ levels were >900 pg/mL after 35 days of r-hFSH stimulation. AFC = antral follicle count; BMI = body mass index; CLD = chronic low-dose; E_2_ = oestradiol; hCG = human chorionic gonadotrophin; LD = low-dose; PP = per-protocol; PRL = prolactin; r-hFSH = recombinant human follicle-stimulating hormone.

Of the 320 patients who were screened, 310 were randomized and received at least one dose of study treatment: 155 patients in each treatment group (Figure 2). Thirteen patients were withdrawn prematurely from the study due to pregnancy before stimulation (*n =* 2), financial reason (*n =* 1), patient decision (*n =* 1) or lost to follow up (*n =* 9). A further 50 patients were excluded from evaluations: 45 due to protocol violations (age-related [*n =* 9], BMI-related [*n =* 30], high FSH [*n =* 1], high prolactin [*n =* 5]) and 5 who were randomized to the wrong treatment. The primary efficacy analysis population comprised 247 patients: 122 patients in the CLD group and 125 in the LD group. Patients’ demographic and baseline characteristics were similar in the two groups (primary efficacy analysis population; Table 1).A total of 189 patients were included in the PP population (95 and 94 patients in the CLD and LD groups, respectively; Figure 2).

**Table 1:** Patients’ demographic and baseline fertility characteristics overall and by treatment group (primary efficacy analysis population)

**Variable**		**CLD group**	**LD group**	**Total**
		**(*n*** **= 122)**	**(*n*** **= 125)**	**(*N*** **= 247)**
*Age, years*^*a*^	*n*	122	124	246
	*Mean (SD)*	27.5 (4.42)	27.9 (4.33)	27.7 (4.37)
*BMI, kg/m*^*2*^^*a*^	*n*	119	124	243
	*Mean (SD)*	26.4 (2.95)	26.0 (3.14)	26.2 (3.05)
*Race*^*b*^	*n*	122	124	246
*Arab*	*n (%)*	68 (55.7)	69 (55.6)	137 (55.7)
*Asian*	*n (%)*	19 (15.6)	16 (12.9)	35 (14.2)
*Black*	*n (%)*	1 (0.8)	4 (3.2)	5 (2.0)
*Caucasian*	*n (%)*	33 (27.1)	34 (27.4)	67 (27.2)
*Other*	*n (%)*	1 (0.8)	1 (0.8)	2 (0.8)
*Infertility type*^*b*^	*n*	122	125	247
*Primary*	*n (%)*	92 (75.4)	96 (76.8)	188 (76.1)
*Secondary*	*n (%)*	30 (24.6)	29 (23.2)	59 (23.9)
*Duration of infertility, years*^*a*^	*n*	122	125	247
	*Mean (SD)*	3.8 (2.57)	4.2 (2.48)	4.0 (2.53)
*Cause of infertility*^*b*^	*n*	123^c^	127^c^	250^c^
*Ovulatory dysfunction*	*n (%)*	120 (97.6)	121 (95.3)	241 (96.4)
*Tubal factor*	*n (%)*	0 (0.0)	1 (0.8)	1 (0.4)
*Other*	*n (%)*	3 (2.4)	5 (3.9)	8 (3.2)
*Previous infertility treatment*^*b*^	*n*	122	125	247
*Yes*	*n (%)*	90 (73.8)	103 (82.4)	193 (78.1)
*No*	*n (%)*	32 (26.2)	22 (17.6)	54 (21.9)
*Previous pregnancies*^*b*^	*n*	122	125	247
*None*	*n (%)*	92 (75.4)	94 (75.2)	186 (75.3)
*At least one*	*n (%)*	30 (24.6)	31 (24.8)	61 (24.7)

^a^Not significant; *t*-test.

^b^Not significant; Chi-square test.

^c^Multiple causes of infertility in three patients (CLD group, *n* = 1; LD group, *n* = 2).

BMI = body mass index; CLD = chronic low-dose; LD = low-dose; SD = standard deviation.

### Efficacy

Similar rates of mono-follicular development (primary endpoint) were achieved in the CLD and LD groups in the primary efficacy analysis population: 56.6% (69/122) and 55.2% (69/125), respectively (p = 0.93; Table 2). This was also the case in the PP population: 64.2% (61/95) and 62.8% (59/94), respectively (p = 0.96; Table 3).

**Table 2:** Primary and secondary efficacy endpoint results in the primary efficacy analysis population

**Efficacy endpoint**	**CLD group**	**LD group**	**p**
	**(*n*** **= 122)**	**(*n*** **= 125)**	
*Mono-follicular development*^*a*^	69 (56.6)	69 (55.2)	0.93
*Bi-follicular development*^*a*^	17 (13.9)	22 (17.6)	0.54
*Multi-follicular development*^*a*^	17 (13.9)	15 (12.0)	0.79
*Cycle cancellation rate*^*b*^	19 (15.6)	19 (15.2)	0.94
*hCG injection rate*	94 (77.1)	91 (72.8)	0.53
*Clinical pregnancy rate*^*c*^	19 (20.2)	18 (19.8)	0.94

Data are *n* (%).

^a^Number of patients with follicles ≥17 mm in diameter by Day 35 of stimulation.

^b^Due to inadequate response.

^c^Pregnancy rate calculated per patients who received hCG injections (CLD group, *n* = 94; LD group, *n* = 91).

p values show the difference between treatment groups, using the Chi-square test.

CLD = chronic low-dose; hCG = human chorionic gonadotrophin; LD = low-dose.

**Table 3:** Primary and secondary efficacy endpoint results in the per-protocol population

**Efficacy endpoint**	**CLD group**	**LD group**	**p**
	**(*n*** **= 95)**	**(*n*** **= 94)**	
*Mono-follicular development*^*a*^	61 (64.2)	59 (62.8)	0.96
*Bi-follicular development*^*a*^	16 (16.8)	19 (20.2)	0.68
*Multi-follicular development*^*a*^	11 (11.6)	10 (10.6)	0.84
*Cycle cancellation rate*^*b*^	7 (7.4)	6 (6.4)	0.79
*hCG injection rate*	84 (88.4)	82 (87.2)	0.98
*Clinical pregnancy rate*^*c*^	17 (20.2)	17 (20.7)	0.37

Data are *n* (%).

^a^Number of patients with follicles ≥17 mm in diameter by Day 35 of stimulation.

^b^Due to inadequate response.

^c^Pregnancy rate calculated per patients who received hCG injections (CLD group, *n* = 84; LD group, *n* = 82).

p values show the difference between treatment groups, using the Chi-square test.

CLD = chronic low-dose; hCG = human chorionic gonadotrophin; LD = low-dose.

No significant differences between treatment groups were found in the secondary efficacy endpoints. Rates of bi-follicular development, multi-follicular development, and hCG administration in the primary efficacy and PP populations are presented in Table 2 and 3. Clinical pregnancy rates for those patients who received an hCG injection were 20.2% (19/94) and 19.8% (18/91) in the CLD and LD groups, respectively (p = 0.94; primary efficacy analysis population; Table 2). The rate of cycle cancellation owing to inadequate response was 15.6% (19/122) of patients in the CLD group and 15.2% (19/125) of patients in the LD group (p = 0.94, primary efficacy analysis population).

In those patients who received an hCG injection, the mean duration of r-hFSH stimulation required for OI, while not statistically significant, was slightly longer in the CLD than in the LD group (13.7 versus 12.9 days, respectively; primary efficacy analysis population; Table 4). In addition, the mean daily and total doses of r-hFSH required were slightly lower in the CLD group versus the LD group, although only mean daily dose showed a statistically significant difference between groups (Table 4 and 5).

**Table 4:** Duration of recombinant human follicle-stimulating hormone (r-hFSH) stimulation and total and daily r-hFSH doses required - primary efficacy analysis population (patients who received hCG injections)

	**CLD group**	**LD group**	**Total**	**p**
	**(n = 94)**	**(*n*** **= 91)**	**(*N*** **= 185)**	
*Duration of stimulation, days*	13.7 +/− 6.33	12.9 +/− 5.58	13.3 +/− 5.97	0.36
*Daily dose, IU*	78.7 +/− 8.90	85.0 +/− 15.35	81.8 +/− 12.86	**<0.001**
*Total dose, IU*	1119.4 +/− 690.04	1155.5 +/− 730.45	1137.2 +/− 708.50	0.73

Data are mean +/− standard deviation.

p values show the difference between treatment groups using analysis of variance.

Values in bold are significant.

CLD = chronic low-dose; hCG = human chorionic gonadotrophin; LD = low-dose.

**Table 5:** Duration of recombinant human follicle-stimulating hormone (r-hFSH) stimulation and total and daily r-hFSH doses required - Per-protocol population (patients who received hCG injections)

	**CLD group**	**LD group**	**Total**	**p**
	**(*n*** **= 84)**	**(*n*** **= 82)**	**(*N*** **= 166)**	
*Duration of stimulation, days*	13.4 +/− 6.04	13.0 +/− 5.81	13.2 +/− 5.91	0.66
*Daily dose, IU*	78.5 +/− 8.95	85.5 +/− 15.88	81.9 +/− 13.28	**<0.001**
*Total dose, IU*	1092.9 +/− 665.19	1183.1 +/− 762.52	1137.4 +/− 714.18	0.42

Data are mean +/− standard deviation.

p values show the difference between treatment groups using analysis of variance.

Values in bold are significant.

CLD = chronic low-dose; hCG = human chorionic gonadotrophin; LD = low-dose.

### Safety

Of the 135 patients included in the safety population, 30/70 (43%) in the CLD group and 31/65 (48%) in the LD group experienced at least one AE, excluding injection-site reactions. In total, 121 AEs were reported (53 in the CLD group; 68 in the LD group). The majority of AEs were mild in severity (106/121; 87.6%), with only 11 (9.1%) moderate and two (1.7%) severe AEs reported (the severity of two AEs was not recorded). There was only one report of OHSS (CLD group), which was mild in severity and resolved without sequelae. However, the patient was withdrawn from the study as discontinuation was mandatory following the development of OHSS.

Only one AE resulted in treatment discontinuation and two resulted in modification of treatment. One serious AE was reported: an ectopic pregnancy (moderate in severity) occurred in a patient in the LD group and was considered to be unrelated to the study drug. The patient was hospitalized and successfully managed with medical treatment (methotrexate). The ectopic pregnancy resolved without sequelae.

No significant difference was found between multiple pregnancy rates in the CLD and LD groups for patients who received hCG: 3.2% (3/94) versus 1.1% (1/91), respectively (p = 0.34), in the primary efficacy analysis population and 2.4% (2/84) versus 1.2% (1/82), respectively (p = 0.58), in the PP population. All multiple pregnancies reported were twins. No significant difference was found between miscarriage rates (for patients who received hCG) in the CLD and LD groups: 4.3% (4/94) versus 6.6% (6/91), respectively (p = 0.48), for the primary efficacy analysis population and 4.8% (4/84) versus 6.1% (5/82), respectively (p = 0.70), for the PP population.

#### Injection-site tolerability

Treatment diaries were provided by 150 patients (CLD group: *n* = 73; LD group: *n* = 77). Of these, 42 patients (28%) reported ≥1 r-hFSH injection-site reaction (CLD group: *n* = 19; 26.0%; LD group: *n* = 23; 29.9%). The r-hFSH injection-site reactions reported were pain (CLD group: *n* = 9; LD group: *n* = 20), redness (CLD group: *n* = 2; LD group: *n* = 5), bruising (CLD group: *n* = 6; LD group: *n* = 2), swelling (CLD group: *n* = 4; LD group: *n* = 1) and irritation (CLD group: *n* = 2; LD group: *n* = 3).

## Discussion

To the authors’ knowledge, the MEnTOR study is the first large, randomized, multicentre trial to compare the efficacy and safety of a CLD versus LD step-up OI treatment protocol using follitropin alfa FbM (r-hFSH). Previously reported data from a small, single-centre study suggested that the LD protocol may substantially reduce the FSH dose requirement and mean treatment duration versus the CLD protocol while maintaining similar pregnancy rates [8]. However, the LD protocol may be associated with a higher multiple pregnancy rate than the CLD protocol [8]. The high batch-to-batch consistency and precise dosing of follitropin alfa FbM was expected to allow any differences in outcomes of the two treatment protocols to be revealed, by reducing individual variability in ovarian response [19]. However, follitropin alfa FbM has been reported to reduce the rate of OI cycle cancellation owing to poor response (p < 0.02) and increase the proportion of cycles not requiring a dose increase (p < 0.001), compared with the follitropin alfa filled-by-bioassay formulation [21]. Indeed, it is possible that the reduced requirement for adjustment of the starting dose when using follitropin alfa FbM [21] could explain the similarity in outcomes of the CLD and LD protocols demonstrated in the current study.

In the current study, the CLD and LD protocols demonstrated similar efficacy, with no differences found in the primary and secondary efficacy outcomes. In addition, the two protocols were similar in terms of safety outcomes. Of particular importance, similar rates of multiple pregnancy (among patients who received hCG) were reported with the CLD and LD protocols (3.2% versus 1.1%, respectively). It should be noted that the LD protocol was associated with a slightly higher mean daily and total r-hFSH dose requirement, but a shorter duration of stimulation versus the CLD protocol.

Rates of clinical (20.2%) and multiple pregnancy (3.2%) for patients who received hCG in the CLD group are in line with a previous compilation of data from 11 CLD protocol studies (21–45% and 0–14%, respectively) [8]. Mono-follicular and bi-follicular development rates reported here in the CLD group are also similar to those reported in two recent publications of similar CLD protocols (54–55% and 17–25%, respectively) [9,16]. Minimal data on the outcomes of LD treatment protocols are available. The multiple pregnancy rate of the LD protocol in the current study was lower than that reported previously [8]; however, direct comparison of data between studies is limited because of differences in the methodology used to calculate rates.

The strengths of the current study include the large randomized population (*N* = 310) recruited from six Middle Eastern countries, and the use of follitropin alfa FbM (rather than a formulation of urine-derived hFSH or r-hFSH calibrated using the Steelman and Pohley bioassay). Follitropin alfa FbM is assayed using a physicochemical analytical method, which ensures a precise content per vial and high batch-to-batch consistency [18].

Identification of factors predictive of response prior to stimulation could also improve the efficacy and safety of OI treatment protocols [16,22]. Such information would allow FSH thresholds for successful ovulation to be better estimated and individualized starting doses defined [16,22]. In an analysis of data from two prospective, randomized, Phase III, multicentre studies, low BMI, low antral follicle count and high normal baseline FSH level were associated with successful OI using a CLD r-hFSH protocol [16]. Previous studies have also identified BMI as a major determinant of successful ovulation, along with cycle history, baseline FSH level, insulin-like growth factor I concentrations and previous history of response to CC [14,23]. Obesity and insulin resistance have been associated with adverse outcomes [14,23].

A potential limitation of the current study is the use of a standard starting dose of r-hFSH regardless of the individual’s BMI (range 20–32 kg/m^2^). It is possible that failure to tailor the initial dose of r-hFSH could have contributed to the lack of difference in outcomes shown between the two protocols.

## Conclusions

This study provides valuable information on the efficacy and safety of LD and CLD protocols used for OI in women with WHO Group II anovulatory infertility in the Middle East. The r-hFSH LD and CLD protocols resulted in similar efficacy and safety outcomes; although the CLD protocol may be more suitable in patients at risk of multifollicular development.

## Abbreviations

AE: Adverse event; BMI: Body mass index; CC: Clomiphene citrate; CLD: Chronic low dose; E_2_: Oestradiol; FbM: Filled by mass; FSH: Follicle-stimulating hormone; hCG: Human chorionic gonadotrophin; LD: Low dose; MEnTOR: Middle East Trial for Ovulation induction Responders; OHSS: Ovarian hyperstimulation syndrome; OI: Ovulation induction; P4: Progesterone; PP: Per protocol; r-hCG: Recombinant human chorionic gonadotrophin; r-hFSH: Recombinant human follicle-stimulating hormone; TVUS: Transvaginal ultrasound; WHO: World Health Organization.

## Competing interests

Gamal I Serour has received a grant from Al Azhar University. Jean-Noel Hugues has participated in previous international studies on products manufactured by Merck Serono. Khaled Esmat was an employee of Merck Serono Middle East FZ – LLC, Dubai, UAE (an affiliate of Merck KGaA, Darmstadt, Germany) at the time of the study. Mohamed Aboulghar and Awatef Al Bahar declare that they have no competing interests.

## Authors’ contributions

All authors provided substantial contributions to conception and design, acquisition of data, analysis and interpretation of data, and revised the manuscript critically for important intellectual content. All authors read and approved the final manuscript.

## Supplementary Material

Additional file 1 CONSORT 2010 checklist of information to include when reporting a randomised trial*. Click here for file
